# Through the Immune Looking Glass: A Model for Brain Memory Strategies

**DOI:** 10.3389/fncel.2016.00017

**Published:** 2016-02-04

**Authors:** Silvia Sánchez-Ramón, Florence Faure

**Affiliations:** ^1^Department of Clinical Immunology and Instituto de Investigación Sanitaria San Carlos (IdISSC), Hospital Clínico San CarlosMadrid, Spain; ^2^Department of Microbiology I, Complutense University School of MedicineMadrid, Spain; ^3^Institut National de la Santé et de la Recherche Médicale U932, Institut CurieParis, France

**Keywords:** hippocampus, innate and adaptive memory, implicit and explicit memory, trained immunity, recognition, immune system, central nervous system

## Abstract

The immune system (IS) and the central nervous system (CNS) are complex cognitive networks involved in defining the identity (self) of the individual through recognition and memory processes that enable one to anticipate responses to stimuli. Brain memory has traditionally been classified as either implicit or explicit on psychological and anatomical grounds, with reminiscences of the evolutionarily-based innate-adaptive IS responses. Beyond the multineuronal networks of the CNS, we propose a theoretical model of brain memory integrating the CNS as a whole. This is achieved by analogical reasoning between the operational rules of recognition and memory processes in both systems, coupled to an evolutionary analysis. In this new model, the hippocampus is no longer specifically ascribed to explicit memory but rather it both becomes part of the innate (implicit) memory system and tightly controls the explicit memory system. Alike the antigen presenting cells for the IS, the hippocampus would integrate transient and pseudo-specific (i.e., danger-fear) memories and would drive the formation of long-term and highly specific or explicit memories (i.e., the taste of the Proust’s madeleine cake) by the more complex and recent, evolutionarily speaking, neocortex. Experimental and clinical evidence is provided to support the model. We believe that the singularity of this model’s approximation could help to gain a better understanding of the mechanisms operating in brain memory strategies from a large-scale network perspective.

## Roads to Memory and The Self

Our current state of knowledge is beginning to grasp the true essence of the evolutionarily developed processes underlying context-specific recognition and memory by the central nervous system (CNS). The CNS, and more particularly the human brain, consists of an extremely complex network of interactions among neural cells. This network coordinates all actions of the organism, intrinsically embodied with abstract thinking and high intellectual functions up to what we know as mind. Meanwhile, the immune system (IS) brings together an extensive network of cells, molecules and organs that are involved in the maintenance of the organism’s integrity, conceptualized by some authors as a real neural network (Varela and Coutinho, 1989, 1991). The CNS and the IS are thus complex integrative systems, showing some intriguingly similar general functions, although at different levels of organization and complexity. Both systems are involved in defining the identity (self) of the individual (in terms of unique genetic and phenotypic attributes), together with continuing cumulative experience (history), through memory.

Memory represents one of the most transcendent properties of the neural and immunological activity, enabling modification and anticipation of responses to stimuli, and also providing a valence or category to each response. The valences can be synthesized as predominantly negative (danger-fear cues inducing effector responses in the CNS and in the IS) or positive (action through reward and tolerance, respectively). Brain memory is the substrate of our intelligence, emotions, evoked feelings and behavior, while immune memory allows efficient clearing of pathogenic microbes and tolerance to diverse symbiotic microbial populations and to the own host cells. Both systems could also be functionally equivalent in their ultimate goal, the maintenance and survival of the organism. Their capability for information processing and proactive responsiveness in the face of uncertainty are the features that define a cognitive system, although at quite different scales.

The CNS and the IS deal with the external and internal reality by direct interaction and recognize the potential stimulus through biochemical and electromagnetic coupling interactions at quite different levels. Through these interactions both systems subsequently generate an internal representation of such reality. A sharp distinction between the CNS and the IS is therefore apparent at first sight. Neurons possess one distinctive characteristic that is their intrinsic oscillatory electromagnetic activity, basis of neural transfer of information, which renders the system able to offer immediate responses. It has been commonplace to consider memory traces as structural modifications on the strength of synaptic connections at neuronal dendritic spines in a phenomenon called long-term potentiation (LTP) and on the establishment of new neural circuitries, mostly based in individual neurons and pathways in what has been called the Neuron Doctrine (Ramón y Cajal, 1891; Hebb, 1949; Kandel et al., 2014; Nabavi et al., 2014). However, the mechanisms of how a given memory is specifically encoded at the molecular and furthermore at high order associative levels remains to be deciphered.

One could object to the analogies between the CNS and IS on the grounds of the current knowledge that such analysis could work for general mechanisms, but have limited application for the specifics of brain memory. Indeed, a major problem is the integration of logical thinking and self-awareness (consciousness) of the neural memory process, which cannot have a counterpart in the IS. Caveats issued, this approximation may provide a valuable opportunity to explain the global functioning of brain memory.

We have worked on the analogies of relationships between the general mechanisms and the subcellular events for memory processes in both systems. Analogies were coupled with an evolutionary analysis of the recognition and memory strategies. Our model generates an apparent paradox: while the terminology of IS processes has been described as a metaphor of their CNS counterparts (with terms such as recognition, learning, priming, memory, tolerance and synapse, among others); we intend here the task to build-up a model of brain memory by applying the conceptual framework of the immune memory system to the brain memory system as an approach to study emergent levels of brain neural networks as a whole (Box 1).

Box 1 **Parallelism between general rules of the IS and the CNS.**

- The IS and the CNS are complex cognitive networks safeguarding the identity (self) of the individual by means of recognition and memory mechanisms throughout lifetime.
- Shared general mechanisms and phenomena in recognition, (learning) and memory are apparent and account for the high diversity, specificity and plasticity of each system.
- Analogical reasoning between operational rules of recognition and memory in the IS and the CNS allows an approximation to some CNS’ emerging and associative properties of the network.

Brain memory has traditionally been classified as either implicit or explicit on psychological and anatomical grounds, with reminiscences of the evolutionarily-based innate-adaptive IS responses. According to this model, we focus on the role of hippocampus and introduce the new concept of its role in controlling both implicit and explicit memory system. Not unlike the antigen presenting cells for the IS, the hippocampus would integrate transient and pseudo-specific (i.e., danger-fear) memories and would drive the formation of long-term and highly specific memories (i.e., the taste of the Proust’s madeleine cake) by the more complex and recent, evolutionarily speaking, neocortex.

## Revisiting The Classification of Recognition and Memory Strategies: Why An Immune Hypothesis?

The existing classification of implicit and explicit brain memory sets out the concepts that underlie the whole multidisciplinary knowledge and research on this intricate yet fascinating issue. However, this classification may show some inaccuracies, due mainly to the multiple and sometimes overlapping memory systems (Willingham and Goedert, 2001; Henke, 2010), and the more descriptive than explanatory taxonomy. Here, we outline some of the most common concerns for which looking through the immune optic might assist to clarify aspects in the current brain memory model:

- *Notions or biases that guide our present knowledge on CNS memory:* the classification of memory types initially was, and still is today, based primarily on a psychological and neuroanatomical perspective (Reber, 2013), and thus could not necessarily align with evolutionarily gained structures. Besides, the classical evolution- and *scala naturae*-based terminology for the brain organization does not always accurately reflect current understanding and this may have blurred the interpretation of the function of certain brain structures (Jarvis et al., 2005).
- *Accelerated parallel evolution of both IS and CNS*. As complex information processing systems, the major force of natural selection pressure along evolution has targeted genes affecting immunity and nervous systems, together with reproduction (Dorus et al., 2004; Barreiro and Quintana-Murci, 2010; Barry and Mattick, 2012).
- *Shared embryogenesis phenomena in both highly integrated systems*, particularly in the education on identity (self) intrauterus; similarities between double-positive thymic T-cells and neuroblasts; shared programmed-cell death (PCD) mechanisms; putative non-homologous end-joining molecules (Chun, 2001); and myeloid origin of the microglia; among others.
- *Primitive elements are highly conserved and remain crucial for immune and neural responsiveness*. According to Medzhitov and Janeway (2002), the major decision to respond to a particular stimulus is primarily decided by the innate IS. The same principle works for the CNS, in which the major decision to respond to a particular stimulus is determinant for survival and corresponds to the most ancient part of the brain. By contrast, the ability to specifically remember a given stimulus in both systems is found exclusively in vertebrates and not in plants or invertebrates.
- *Specific (or explicit) memory is associative and remains preserved by a specialized dispersion in the complex network*. The immune input contains multiple specific determinants (or epitopes) that shape their multiple cognate cells’ and antibodies interactions in coordinated immune outputs. Alike, brain memories are encoded as synchronous multineuronal activation patterns and not at a single cell level (Yuste, 2015).

Additional common shared characteristics of memory processing in both systems that might show archetypal principles, are: (i) the duality of specificity and degeneracy of recognition, favoring the precision and plasticity of response; (ii) experience-dependent plasticity, as repetitive encounters reinforce the memory process inducing multiple alternative pathways for any given element; (iii) redundancy in mechanisms, more common in the innate immune and CNS for fail-safe operations, since they impact directly on survival; and (iv) feed-back loops for homeostatic regulation of the system. These parallelisms have experimental evidence in pathology, as we shall illustrate.

## Evolutionary Framework for Memory Classification

The classification of innate and adaptive IS has been based on an evolutionary perspective, showing distinct definite mechanisms of recognition and memory and timeline of action. This view has important implications for the understanding of immune mechanisms of memory. Innate recognition mechanisms are highly conserved across phyla, driving the specific adaptive immune responses in higher vertebrates (Sánchez-Ramón and Butnaru, 2013). Meanwhile, adaptive memory strategies correspond to evolutionarily innovated and biochemically distinct highly diverse specific receptors capable of generating a more rapid, efficient and sophisticated response through memory. Therefore, innate control of adaptive immunity is now a well-established paradigm (Iwasaki and Medzhitov, 2015), which warrants the regulation of a more complex level of immunity in terms of economy and avoidance of potentially harmful responses.

In contrast, the present-day classification of two major types of CNS memory in implicit and explicit stems from its psychological content—perceptual and motor skills and without awareness vs. conscious contextual and semantic; and from its anatomical involvement—non-medial temporal lobe (MTL)-mediated vs. MTL-mediated—(Scoville and Milner, 1957, 2000; Graf and Schacter, 1985; Squire, 1992). Thus, MTL (hippocampus and adjacent structures) has been to date ascribed to explicit memory, what we are going to challenge here. Implicit and explicit types of memory have been classically described as quite independent of each other (Graf and Schacter, 1985; Schacter, 1987), which are referred to as dissociation and show neuroimaging correlates (Korsnes and Magnussen, 2014).

By analogy with the innate control of adaptive IS, we postulate that MTL is part of the innate (implicit) system and exerts control of explicit memory. Indeed, MTL homolog structures exist across invertebrate taxa, such as the mushroom bodies of arthropods (Kandel and Abel, 1995; Wolff and Strausfeld, 2015). Many areas of complex behavior rely on brain systems that are separate but operate in cooperative fashion. Consequently, MTL may have a dual role as seat for implicit memories and in giving rise to explicit ones. By contrast, the neocortex is unique among higher vertebrates and has evolved independently in humans (Karten, 1991; Roth and Dicke, 2005, 2012; Wang et al., 2010; Bruner and Iriki, in press) and hence, we can conjecture that the neocortex might account for the development of explicit memory, as we are going to explain below in a more in-depth way (Box 2).

Box 2 **Overlay of classification of recognition and memory from the IS to the CNS.**

- Innate and adaptive immunity support different strategies and molecular solutions of recognition and memory acquired during evolution, which fit with differential quality and duration of the memory.
- Accordingly, we propose that implicit (perceptual and motor skills without awareness) and explicit (conscious contextual and semantic skills) memory should be explained by different strategies and molecular solutions.

The pioneering seminal discovery of the protein substrate for implicit memory consolidation in the sea slug *Aplysia californica* corresponds to the cytoplasmic polyadenylation element binding protein or CPEB (Si et al., 2003a). CPEB is involved in the specificity and strengthening of the synapse, showing functional self-perpetuating prion-like properties (Si et al., 2003b). This protein seems to be highly conserved through phyla for noxious memory traces in neural circuits in both invertebrates and vertebrates including the human brain (Kandel et al., 2014). Prion-like proteins have also been ascribed in explicit memory circuits (Barco et al., 2006), and have been suggested as common molecular mechanisms to both implicit and explicit memory types. However, we may hypothesize that distinct alternative evolutionary processes have developed from the proteins specialized in innate memory processes to the specific-content-discriminating memories in the vertebrates’ brain. MTL is necessary but not sufficient for explicit memory consolidation, as MTL lesions have shown to abrogate newly acquired memories and learning but not previously acquired long-term consolidated memories (Scoville and Milner, 1957, 2000).

On the other hand, a recent revision of the 100-year-old brain anatomy classification by using gene-expression and embryology studies have enabled more accurate evolutionary brain models that has entirely changed its functional interpretation. In fact, an agreed amendment of the anatomical nomenclature of the avian brain has lead to a divergent analysis of much more complex cognitive abilities of some brain regions than previously appreciated. In particular, the nuclear avian *pallium* becomes to be considered homolog to the six-layered cortex or neocortex in mammals (Jarvis et al., 2005). The authors state the necessity of a re-evaluation of different neuroarchitectural solutions between avians and mammals to interpret sophisticated cognitive behaviors and to favor the emergence of new insights.

Altogether, we believe that the integrated model of innate and adaptive immune mechanisms may provide a conceptual framework for studying the roles of comparable neurobiological strategies of recognition, learning and memory.

## Integrating Innate and Adaptive Recognition and Memory Strategies in Immune and Nervous Systems

### Innate or Implicit Strategies

The IS and the CNS are extremely efficient systems along ancestral innate strategies from invertebrates to higher vertebrates. In the IS, the recognition strategies are either conserved throughout evolution or the result of convergent evolution in insects and vertebrates, as recently suggested for some toll-like receptors (TLRs; Hughes, 1999; Luo and Zheng, 2000; Kanzok et al., 2004). The traditional paradigm stresses the view that our innate IS encompasses mechanical barriers, secretions and soluble proteins at the front-line of host defense, as well as a large set of receptors that sense for exogenous and endogenous signals. Largely invariant shared microbial molecules are recognized by pathogen pattern recognition receptors (PRRs; Table 1). These interactions can induce danger signals and cellular stress, initiating the effector immune response (Gallucci and Matzinger, 2001; Janeway and Medzhitov, 2002; Hoffmann, 2003; Akira et al., 2006); or signals leading to the tolerogenic response (Cavaillon et al., 2003; Li et al., 2006). PRRs include TLRs, nucleotide-binding oligomerization domain (Nod)-, leucine-rich repeat–containing receptors (NLRs), RIG-I-like receptors (RLRs), C-type lectin receptors (CLRs) and AIM-2 like receptors, as well as intracellular sensors of nucleic acids (Thery and Amigorena, 2001; Iwasaki and Medzhitov, 2015). Interestingly, these ligand-receptor interactions occur within a range of affinity and also multiple specificities, highlighting the biologic relevance of competitive and regulatory mechanisms (Miyake, 2007; Kondo et al., 2012). For instance, TLR4 can bind to bacterial lipopolysaccharides (LPS), but also to cellular hsp70 and hyaluronan (Matzinger, 2002). Despite some putative change of sensitivity status when second messenger accumulates (e.g., cyclic GMP–AMP synthase or cGAS products), no memory is ascribed to this line of strategies, which could be defined as intrinsic immunity (Table 1). Finally, these PRRs are also present in cells of the adaptive system, as a result of co-evolution.

**Table 1:** **Proposed parallel classification of recognition and memory strategies**.

IS	Innate		Adaptive
	Intrinsic	Trained IMM
Response timeline	Immediate	Immediate	Delayed, regulated by the innate IS
Recognition/memory	Non specific (no memory)	Pseudo-specific, memory-like transient modifications	Specific, long-term memory
Distribution	Soluble molecules, non- hematopoietic cells, immune cells (monocytes/macrophages, DCs, ILCs, NKT, γδT cells), neutrophils, basophils, eosinophils	NK cells, monocytes/macrophages, DCs ILCs, NKT, γδT cells	B cells, Ab, T cells
Mechanisms of recognition/imprinting	Physical barriers, soluble proteins PRRs and DAMPs: receptors fixed in the genome	PRRs and DAMPs, semi-specific PRRs: receptors fixed in the genome Epigenetic reprogramming	TCR, BCR: gene rearrangement (diversity repertoire). Epigenetic reprogramming
Type of response	Stereotyped	Enhanced efficiency after re-exposure categorization	Clonal, secondary response
**CNS**	**Implicit**		**Explicit, declarative**
	**Intrinsic**	**Trained (Working memo)^£^**
Response timeline	Immediate	Immediate^£^	Delayed, regulated by the implicit memory
Recognition/memory	Not specific, unconscious	Pseudo-specific, pseudoconscious, transient memory^£^	Highly specific, long-term memory
		Primary consciousness^£^	High order consciousness
Distribution	Primary and secondary-modality specific cortex. Brainstem, hypothalamus, cerebellum	Amygdala and basal ganglia^£^ MTL^£^ Rostral anterior cingulate cortex^£^	Prefrontal cortex, posterior parietal cortex (neocortex) ^£^
Mechanisms of recognition/imprinting	Receptors fixed in the genome	Prion-like proteins^£^ Engram cells persistent connectivity^£^ Epigenetic reprogramming^£^	Diverse repertoire of recognition structures for specific gamma-band spikes? ^£^ Epigenetic reprogramming
Type of response	Stereotyped (instinctive, automatisms)	Experience-dependent changes^£^ Categorization^£^ Special-purpose tasks^£^	Associational tasks, general purpose tasks^£^

*Ab, antibody; BCR, B cell receptor; DAMPs, Damage-associated molecular pattern molecules; DC, dendritic cells; ILCs, innate lymphoid cells; NKT, Natural Killer T cells; MTL, medial temporal lobe (hippocampus, entorhinal cortex, perirhinal, parahippocampal); PRR, pattern recognition receptors; TCR, T cell receptor. ^£^Categories and features proposed in the model*.

A high degree of genetic degeneracy and redundancy (Edelman and Gally, 2001) together with acquired compensation mechanisms in the innate IS warrants survival. In fact, deficiencies of any PRRs result very rarely in life-threatening infections. Exceptions are the congenital TLR3-TRIF pathway deficiency associated with herpes simplex virus encephalitis (Casrouge et al., 2006); and the MyD88- and IL-1R associated kinase (IRAK)-4-dependent pathway that is essential for protective immunity to a few pyogenic bacteria in childhood but otherwise redundant in host defense (Casanova et al., 2011). An example of mechanisms of acquired compensation in this latter IRAK-4 deficiency displaying abolished antiviral TLR-7, -8 and -9 responses is illustrated by the maintenance of normal resistance to virus through TLR-3 and -4 (Yang et al., 2005).

Similarly, the CNS encompasses a large set of multiple sensor modalities with an extremely wide variety of receptors (visual, auditory, olfactory, gustative, kinaesthetic and somatosensory) that converge to primary and secondary sensory cortex and to the brainstem. These interactions could be operating in terms of threats or rewards conferred by external and internal environmental signals. Examples of convergent evolution within the CNS have been reported in invertebrates and vertebrates for olfactory and vision organs (Eisthen, 2002; Ogura et al., 2004; Gehring, 2014) or even in shared mechanisms of cell memory signaling for the IS and the CNS, such as NFkB (Friedman and Hughes, 2002; Lindsley et al., 2014). In analogy to the IS pattern, we could call it as innate intrinsic sensing strategy, giving rise to unconscious stereotyped or automated responses.

Synaptic neural plasticity exemplifies degeneracy in its full meaning (Edelman and Gally, 2001). A high degree of degeneracy, redundancy and dispersion in the implicit memory has been explained in terms of resonance in the network (Reber, 2013). For instance, genetic degeneracy under the lack of sensory receptors is apparent in subterranean mammals such as the blind mole rat, functionally blind and with poor auditory sensitivity but extremely efficient for spatial orientation thanks to a specialized self-generated echolocation system (Kimchi et al., 2004). As an example for acquired compensatory adjustments, the lack of specific sensory nervous receptors, as in human blindness, is associated with compensatory hypertrophy of other sensory modalities (Théoret et al., 2004) and with significant increase in the cerebellar areas coordinating sensory-motor interactions to compensate for the lack of visual information (Lepore et al., 2010).

### “Trained” Memory Strategies

The traditional classification of two, i.e., innate and adaptive IS, from an evolutionary perspective has recently being challenged by the discovery of a transient pseudospecific memory within the innate IS, which represents a paradigm shift in immunity (Netea et al., 2015). Indeed, it is becoming increasingly acknowledged that cells of the innate IS can adapt following a previous encounter with a pathogen in what has been called “trained immunity” (Netea et al., 2011). Invertebrates have proven enhanced secondary immune responses in the absence of adaptive IS (Faulhaber and Karp, 1992) and the capability to transmit unspecific protection to their offspring against original and unrelated pathogens (Rheins et al., 1980). Mammalian prototypical innate immune cells such as NK cells (O’Leary et al., 2006; Sun et al., 2009) or monocytes (Quintin et al., 2012) elicit rapid responsiveness on second encounters, prolonging their survival and self renewal. This trained immunity is antigen-pseudospecific and transient (Netea et al., 2015).

A similar capacity of such pseudospecific transient or “trained” memory may be observed in innate CNS structures, as can be inferred from the following facts (Box 3): (i) evidence for invertebrate brain memory for noxious cues within the model of *Aplysia* in the absence of explicit memory exists. The molecular mechanisms of this long-lasting synaptic plasticity in *Aplysia* and in the mammalian hippocampus (MTL) have proven to have many similarities (Pittenger and Kandel, 2003), and involve the regulation of prion-like protein synthesis at specific dendritic spines; (ii) Hippocampus-like structures called mushroom bodies are present in various invertebrates like bees, insects that exhibit cross-sensory integration, associative learning and memory storage and retrieval. This memory is necessary for the insect to locate multiple places in the environment, as well as for social life (Menzel, 2014); and (iii) Several empirical evidences support the involvement of MTL on implicit and unaware learning tasks besides its undeniable link to explicit memory (Hannula and Greene, 2012; Reber, 2013). Supporting this concept, the works of Dragoi and Tonegawa (2013) have unveiled how fear (innate) contextual cues activate selective ensembles or engrams of hippocampal neurons that contain pre-representations of the external world by specific temporal theta firing sequences in mice. In contrast, high gamma synchrony correlates with explicit (adaptive) awareness of spatial working memory (Yamamoto et al., 2014). Recall for fear contextual memories in mice is primarily based on the re-activation of the same engrams of hippocampal cells activated during initial encoding. This suggests that the memory would reside in these retained specific patterns of connectivity, whereas the strengthening of synapses would be driving the access to activation of such engrams, given that protein synthesis inhibitor did not abrogate engram activation (Ryan et al., 2015). These results are in line with the view that the hippocampus (MTL) is critically involved in implicit fear-conditioned memories, and that its danger-fear valence may be manipulated by concomitant positive stimulus (Redondo et al., 2014). On the other hand, human functional neuroimaging studies have shown the activation of MTL immediately after termination of a new stimulus, referred as hippocampal offset-locked response, predicting the registration of the episodic memories (Ben-Yakov and Dudai, 2011; Ben-Yakov et al., 2013).

Box 3 **Facts further supporting MTL as innate CNS structure.**

- Evidence for regulation of prion-like protein synthesis at specific dendritic spines after noxious cues in invertebrates and vertebrates (MTL).
- Mushroom bodies are hippocampus-like structures in invertebrates like bees that coordinate cross-sensory integration, associative learning and memory storage and retrieval for up to several weeks.
- Cumulative empirical evidence on the role of MTL in implicit as well as in explicit memory.

### Control of Adaptive Memory by Innate Memory Strategies

**Figure 1:**
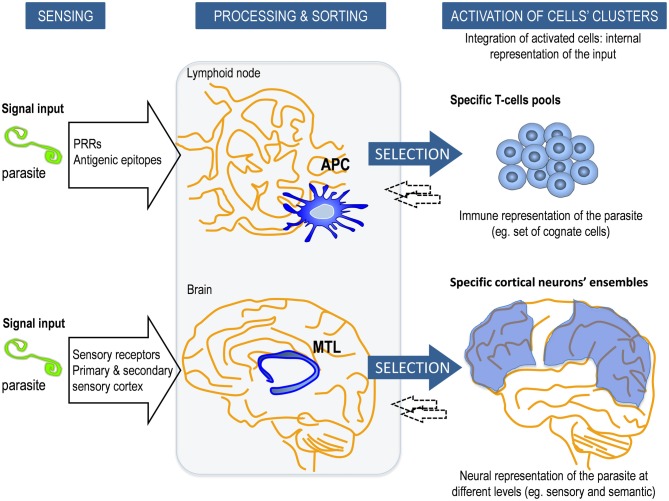
**Schematic model of the innate control of adaptive memory strategies in the immune system (IS) and central nervous system (CNS)**. In adaptive/explicit responses, the long-term memory consolidation requires prior processing and sorting by intermediary innate elements. Our relationship with the external or internal cues is identified, processed and categorized by i.e., antigen-presenting cells (APCs) in the IS and by the medial temporal lobe (MTL) in the CNS. This response is modulated by the nature of the stimulus, their internal status and the local microenvironment context, as well as inputs from other cells within a dynamical framework. APCs (for example, dendritic cells, DC) process antigens into peptides that are presented by major histocompatibility complex (MHC) class I and class II molecules to selected Ag-specific T cells from a vast repertoire. Similarly, MTL is involved in the processing, categorization and transfer of sensory and somatic conscious contents into explicit long-term memories to specific ensembles of neocortical neurons. Reciprocal interactions (←) between them (APC-T-cell and MTL-cortical neuron) occur to adjust the response. The entire ensemble of activated specific cells in each system constitutes the internal representation of the input, which is an emergent process that goes beyond individual cells or components of the network.

As previously stated, the initiation of the adaptive immune response depends on the triggering of innate receptors such as PRRs on the antigen-presenting cells (APCs). APCs such as dendritic cells (DC) are short lived cells specialized in the presentation of antigens to lymphocytes and in the priming the adaptive immune response (Figure 1). These PRRs categorize the origin of the antigens recognized by antigen-specific T and B cells and define the type of response. Consequently, lymphocytes are instructed to differentiate into the appropriate effector class of the immune response depending on the context of presentation (Iwasaki and Medzhitov, 2015). In this setting, specificity but also a certain degree of degeneracy for essentially any antigen can act as a big source of information that can be used freely to adopt enormous numbers of alternative responses. As an advocacy fact supporting this concept, defective signaling of particular TLRs in APCs have been ascribed to impaired recognition and memory responses in prototypical adaptive immune pathologies, namely in autoimmune diseases; and even in congenital immunodeficiencies, such as common variable immunodeficiency (Yu et al., 2009; Mills, 2011).

In our model, this archetypal APC-dependance referred to the human CNS implies the existence of structures *a priori*, namely the hippocampus and adjacent areas (MTL), which would act as the biological substrate of categorization, learning and first step for explicit memory (Figure 1 and Box 4). This *a priori* structure conditionally underlies to human reason in what Kant (1998) defined as the transcendental. It is through such categories that we conceive and think objects. Similarly to what has been described for the APCs, hippocampal cells are in constant renewal and show complex topographical and functional specialization.

Box 4 **Role of the MTL: overlay with an immune system paradigm.**

In parallel with the paradigm of the innate control of adaptive immunity, here we present an immunological-based model for brain memory:

- APCs have the capability to form transient and pseudospecific memory upon encounter with the antigen (trained memory); and drive the specific T and B responses.
- MTL can integrate transient and pseudospecific memory and drive the formation of long-term explicit memory.

### Adaptive or Explicit Strategies

The ubiquity, diversity and rapid co-evolvability of pathogens and host require more complex mechanisms of response. A clonal exceedingly diverse anticipatory repertoire in which each lymphocyte bears a unique antigen-binding receptor—T cell receptors (TCRs) or B cell receptors (BCR or immunoglobulins)—is the central feature of the adaptive IS that evolved in the vertebrate ancestors (Cooper and Alder, 2006). The genes encoding the TCR and BCR are assembled from variable and constant fragments through recombination-activating genes (RAGs) protein-mediated somatic recombination, which yields an extensive variation in the repertoire of receptors (BCR with 10^11^ potential combinations and TCR with 10^16^). Mechanisms such as non-template nucleotide addition, gene conversion and somatic hypermutation for B cell further increase the diversity and specificity in adaptive immune recognition.

In parallel with what has been described for the IS, brain highly specific recognition refers to discrimination of what-is-it type of data characterized by extreme precision and accuracy to the memory. This relies on selection process operating among the diverse repertoire of populations of neurons and on globally coordinated brain activation or consciousness. Current evolutionary theories on human cognitive processes highlight the relevance of the conjoint parieto-frontal neocortex system (Vakhtin et al., 2014) and the *precuneus* (“the eye of the self”; Freton et al., 2014; Bruner and Iriki, in press). Despite an extensive search for DNA rearrangement in the CNS, no locus has been identified to date. Given the highly precise topographical functional organization of brain cells, the actual location of a single neuron contributes to its virtually unique identity (Shatz, 1996; Chun, 2001). The neocortical neurons are immensely diverse (2.1–2.6 × 10^10^; Pelvig et al., 2008). A single cortical neuron can, not only receive inputs from a single cell, but rather a huge number of synapses that has not been uniformly estimated, varying from 5000 to 10,000 (Tang et al., 2001) to up to 30,000 (Rockland, 2002), strikingly comparable ciphers to those described for Ig and TCR. How they are selected for a specific neural signal is still controversial and the structural components of the specific memory remain an enigma.

Consider the specialization of neurons in the visual cortex: feature detection yields a huge diversity of specific patterns that match their receptive fields (Moser et al., 2014). Visual stimuli recruit intrinsically generated cortical circuits of neurons (ensembles) during embryogenesis prior to visual experience as a result of spontaneous activity generated in the retina (Shatz, 1996; retinal and cortical waves). An alternative proposed process involves gap junctions coupling of clonally related neurons at prenatal developmental stage (Li et al., 2012). In both cases, a selective competitive process requiring the formation and elimination of synaptic connections takes place (Shatz, 1996). Strikingly, patients with congenital blindness due to Leber’s congenital amaurosis (LCA) who underwent gene therapy of retinal receptors showed a similar organization of visual circuitries and areas after first visual experience to those in healthy subjects (Ashtari et al., 2015).

According to this view, we can hypothesize that the singularity of the synapse in a neocortical neuron for a specific cue would reside in its specific receptivity for a given codified oscillatory signal sent from the MTL; as long as the singularity of the T-lymphocyte resides in its specific recognition of the peptide presented by the APC (Box 5). In this regard, gamma-band activity has been widely reported to be involved in the cognitive process in general (Varela et al., 2001). An alternative but not exclusive hypothesis advocates that very long-term memories are stored as the pattern of holes in the perineuronal net (Tsien, 2013).

Box 5 **Parallelism between complex processes of adaptive/ explicit memory in the IS and CNS.**

- Selection processes operate in populations of specific cells of each adaptive system that globally and coordinately contain a dynamic representation of the stimulus.
- Adaptive systems rely on highly diverse anticipatory repertoires for specific recognition in spatiotemporal coordinated responses.
- Specific sensitivity to a given codified oscillatory signal from the MTL might provide the singularity of the synapse in a neocortical neuron for a cue.

Moreover, specific recognition is possible without intermediary elements. That is the case of Ig or BCR, capable of recognizing conformational epitopes of antigens. In the CNS, direct route bypassing MTL for explicit semantic memories has been described recently in anterior temporal cortex, a model for associative memory (Merhav et al., 2015). This pathway is used for vocabulary learning by children, and also by amnesic patients with severe MTL lesions (Merhav et al., 2015). Both alternative pathways represent a rapid and direct specific response.

### Emergent Processes in Complex Networks

Adaptive mechanisms generate emergent processes, characteristic of complex systems as a whole, which do not exist in each individual element. For instance, the dynamical behavior of the immune network display non-random patterns of local connectivity of the immune cells coupled with large-scale functional organization. Connectivity at distant sites is accomplished by the migratory capacity of immune cells, in a coherent coordinated response. Similarly, connection patterns of the cerebral cortex typically consists of local and long-range pathways, which renders the system extremely efficient, in what has been called small-world structure (Watts and Strogatz, 1998; Achard et al., 2006).

We broach here how these diverse anticipatory repertoires are modeled by experience through epigenetic marks, evolutionarily-conserved processes that bridge between the IS and the CNS. Epigenetic marks are crucial to normal development (Fan et al., 2001), since these self-perpetuating modifications of gene expression are stably inherited by daughter cells after cell division, contributing to cellular identity despite constant molecular turnover (Turner, 2002). Epigenetic marks account also for the generation and stability of short-term (trained immunity) and long-term (adaptive immunity) memory of novel as well as of recall antigens (Youngblood et al., 2013; Saeed et al., 2014), evoking the concept of recontextualization of the memory. Epigenetic mechanisms operating in brain memory consolidation have been only recently proposed (Day and Sweatt, 2010). Diverse epigenetic mechanisms seem to be responsible of the permanence of synapse-specific changes, including enzyme-catalyzed histone modifications and noncoding RNAs (microRNAs, piwi-interacting RNAs), among others (Guan et al., 2002; Joilin et al., 2014).

## Recall: A Journey into The Past

Retrieval or recall requires link of current cues to past history memory imprintings within a precise spatial cell interaction context. An ensemble of antigen-specific memory T lymphocytes’ repertoire to different epitopes of a particular pathogen or tumor can be retained for a lifetime, implying an heterogeneous selection for multiple high-affinity T-lymphocyte pools that changes over time (Faure et al., 1998). The hallmark of adaptive IS memory is a faster and stronger response upon reencounter with a known antigen. APCs are critical intermediary elements also in recall responses. The site and time-lapse of immune stimulation are key factors for obtaining the appropriate memory and effector responses (Huard et al., 1994) as exploited for immunotherapy design (Zitvogel and Faure, 1999). The exact mechanisms underlying time and location-depending equations are still not well known. However, real-time visualization studies have revealed the differential behavior of antigen-experienced and naïve T lymphocytes following activation and the relevance of coordinated bidirectional communication with innate immune cells (Chtanova et al., 2009; Hor et al., 2015; Luu and Coombes, 2015).

In parallel, immune recall finds its echoes in brain recall mechanisms as a whole. Brain recall entails the activation of an extensive cortical brain network of specific neurons (Ryan et al., 2001; Cabeza et al., 2008). Again, the role of hippocampus in explicit recall is intriguing (Goshen et al., 2011). The hippocampus seems to be normally required for activation of neocortical memory traces depending on the quality of the memory. Reciprocal recursive and reverberating interactions are essential: the prefrontal cortex seems to facilitate top-down selection and organization of retrieval content (Shimamura, 2011), as well as updating relevant features; while the parietal cortex interacts with the MTL and the prefrontal cortex (Cabeza et al., 2008). Aspects of the same memory cut across all levels of hierarchy (semantic, lexical, phonetical, image), each level holding distinct degrees of detail through different time spans. This also evokes the relational nature of memory, not only of individual items but also of compositions of them in associative memory.

Brain lesion magnitude and temporal precision are crucial for remote recall compensation, whereas recent memory relies on hippocampal memory traces (Goshen et al., 2011).

## Conclusions

The methodological standpoint used here enables to approach as a whole the large-scale of CNS network complexity and those emergent processes arising from interactions among multiple neurons and neural circuits. Besides, bringing together the different biological, psychological, clinical and evolutionary data opens a holistic dimension of CNS memory. This approach might help to overcome the inherent difficulty of such a complex system like the human brain being studied by itself. Still, mind transcends any reductionist view of the brain system.

Theoretical models are important tools for many aspects of scientific activity. Our model challenges the traditional classification of brain memory based on a psychological and anatomical perspective. It could reconcile the previous concepts with that of evolutionary and functionally equivalent homologies. This immune-based classification could provide new insights to the established psychological concepts inferred from clinical and behavioral data. The model emphasizes the need of a more accurate categorization of the quality of memories according to the different mechanisms and in an evolutionary context. A major advance has been achieved on the nature and mechanisms underlying innate memories (for fear-contextual memories), which account for specific and persistent engrams cells connectivity at the MTL (Ryan et al., 2015). Our present model supports the view that MTL, as APC for the IS, represents part of the innate memory strategies and a necessary step to adaptive or explicit memory type, instead of what has been to date the mainstream of neuroscientific thought (Scoville and Milner, 1957; Eichenbaum et al., 2007). The proposed classification leaves the neocortex as an evolutionarily acquired highly specific and permanent memory with an open question about the essence of its substrate. In line with our hypothesis, recent findings indicate divergent structure and turnover regulation in hippocampal and neocortical synapses that match with their temporal patterns of memory (transient vs. stable, respectively), suggesting differentially specialized structures in both systems (Attardo et al., 2015). In this study, glutamate receptor blockade contrarily impacted hippocampal and neocortical memory connectivity (Attardo et al., 2015).

In contrast to the accepted dissociation of implicit and explicit memory types by which MTL makes part of the explicit system (Graf and Schacter, 1985; Schacter, 1987), our current model defines however that explicit memory is tightly regulated by the implicit (MTL) system. We explain here this regulation not as a constraint imposed by the system but rather as an evolutionary-emerged property, further modulated in the setting of a historical process (referred to the interplay of genetic, epigenetic and developmental influences). Categories of adaptive responses are predefined by these intermediary elements (MTL and APC) prior to the relation with the object, acquired during embryogenesis and modified by experience all along lifetime. This is compatible with the Kantian concept of human reason, which postulates that our brain does not reflect the world outside but instead it is the world that reflects our brain. For example, the flavor of the chocolate is not in the chocolate itself but in its interactions with our taste buds and sensory areas and the way we perceive it. The same could be inferred for our IS, the immune world is the representation of the interactions of antigens with the immune architecture. Upon this tightly regulated architecture, experience-dependent plasticity through indelible epigenetic mechanisms take place, Further on, as intuited by Heidegger (1962) when analyzing the fundamentals of our reason and his reading on Kant, human understanding acquires its full sense within the dimension of temporality. Altogether, epigenetics could be comprehended as the environmental signature on the individual uniqueness or, in philosophical terms, the source of improvisation (Malabou, 2014).

In both evolutionary acquired adaptive systems, experience is faced with exceedingly diverse pre-formed anticipatory elements for specific recognition in form of patterned associative responses, which in turn establish memory encoding and retrieval in both systems. Spatial temporal precision of interaction of cells ensembles within the network is essential in determining the response and requires bidirectional communications between innate/implicit and adaptive/explicit systems. Not paradoxically, artificial cognitive systems have been built as a powerful metaphor of the natural CNS and IS principles (pattern recognizer, classifier systems based, dynamic learning, error tolerance, anomaly and intrusion detection algorithms, robustness; Jacobsen et al., 2011; Fernandez-Leon et al., 2014).

## Concluding Remarks

The neuron doctrine deals with the first order of approximation to the functioning of the brain that neglects associative patterns and emergent processes of the high order of neural network. By applying analogical reasoning between relationships of the IS and the CNS, we can hold the frame of high order interactions within an evolutionary context. This model leads to the concept that MTL integrates the innate or implicit memory system, giving rise to pseudo-specific memories, and controls the more complex and evolutionary recent neocortex, able of highly specific or explicit memories. We believe that this immune-based model and the new concepts derived from it could inspire the advent of new conceptualizations of large-scale brain networks and empirical approaches towards a unified theory of brain memory.

## Author Contributions

SS-R and FF have equally contributed to the conception and design of the manuscript, drafting, critical revision and final approval of the article. SS-R and FF agree to be accountable for all aspects of the work in ensuring that questions related to the accuracy or integrity of any part of the work are appropriately investigated and resolved.

## Conflict of Interest Statement

The authors declare that the research was conducted in the absence of any commercial or financial relationships that could be construed as a potential conflict of interest.
